# PID-1 is a novel factor that operates during 21U-RNA biogenesis in *Caenorhabditis elegans*

**DOI:** 10.1101/gad.238220.114

**Published:** 2014-04-01

**Authors:** Bruno F.M. de Albuquerque, Maartje J. Luteijn, Ricardo J. Cordeiro Rodrigues, Petra van Bergeijk, Selma Waaijers, Lucas J.T. Kaaij, Holger Klein, Mike Boxem, René F. Ketting

**Affiliations:** 1 Institute of Molecular Biology, D-55128 Mainz, Germany;; 2 Graduate Program in Areas of Basic and Applied Biology, University of Porto, 4099-003 Porto, Portugal;; 3 Hubrecht Institute-KNAW, 3584 CT Utrecht, The Netherlands;; 4 University Medical Center Utrecht, 3584 CT Utrecht, The Netherlands;; 5 Division of Developmental Biology, Department of Biology, Utrecht University, 3584 CH Utrecht, The Netherlands

**Keywords:** 21U, 22G, *C. elegans*, Piwi, biogenesis, piRNA, pid-1

## Abstract

The Piwi–piRNA pathway represents a small RNA-based mechanism responsible for the recognition and silencing of invading DNA. Biogenesis of piRNAs (21U-RNAs) is poorly understood. In *Caenorhabditis elegans*, the piRNA-binding Argonaute protein PRG-1 is the only known player acting downstream from precursor transcription. From a screen aimed at the isolation of piRNA-induced silencing-defective (Pid) mutations, we identified, among known Piwi pathway components, PID-1 as a novel player. PID-1 is a mostly cytoplasmic, germline-specific factor essential for 21U-RNA biogenesis, affecting an early step in the processing or transport of 21U precursor transcripts. We also show that maternal 21U-RNAs are essential to initiate silencing.

## Abstract (precis)

The Piwi–piRNA pathway represents a small RNA-based mechanism to silence invading DNA. It is unknown how transcripts are selected for generation of 21U-RNA, the piRNA class in *C. elegans*. Ketting and colleagues identify PID-1 as a 21U-RNA biogenesis factor and show that PID-1 affects an early step in the processing or transport of 21U precursor transcripts. The authors further show that maternal 21U-RNAs are essential to initiate silencing. This work provides novel insights into piRNA-induced silencing and small RNA biogenesis.

One of the challenges faced by germ cells is the faithful inheritance of their genomes over generations. An important aspect of this process is to ensure that potentially harmful DNA sequences are not expressed and/or do not replicate. Such sequences include transposons and retroviruses and, when left unchecked, create havoc, leading to fertility problems. One of the mechanisms controlling this line of defense is the Piwi–piRNA system (Ghildiyal and Zamore 2009; Malone and Hannon 2009; Luteijn and Ketting 2013).

The Piwi–piRNA pathway is a small RNA-based mechanism. At its core, Argonaute proteins from the Piwi subfamily use small RNA cofactors called piRNAs to recognize and silence transcripts derived from repetitive elements. Silencing often involves the amplification of a small RNA population and can occur at both the post-transcriptional and transcriptional levels. Post-transcriptional silencing involves cleavage of targeted mRNA molecules by the RNase H activity of Piwi proteins, while transcriptional silencing likely involves the recognition of a nascent transcript and is accompanied by the acquisition of repressive chromatin marks at the targeted locus (Ashe et al. 2012; Buckley et al. 2012; Luteijn et al. 2012; Shirayama et al. 2012; Sienski et al. 2012; Huang et al. 2013; Le Thomas et al. 2013; Rozhkov et al. 2013). This heterochromatin-like state can spread and lead to the silencing of genes that are located close to a Piwi target (Sienski et al. 2012). Despite the fact that a number of proteins have been linked to these activities (Ghildiyal and Zamore 2009; Malone and Hannon 2009; Luteijn and Ketting 2013), the exact mechanisms of this transcriptional silencing mechanism remain unclear.

Although the core principles of Piwi-driven mechanisms appear to be strongly conserved, the precise mechanisms through which Piwi proteins act and piRNAs are produced are remarkably flexible between species. In the nematode *Caenorhabditis elegans*, the Piwi protein PRG-1 is guided to its targets by piRNAs (named 21U-RNAs in *C. elegans*) (Ruby et al. 2006; Batista et al. 2008; Das et al. 2008; Wang and Reinke 2008) in a process that tolerates significant mismatches (Bagijn et al. 2012; Lee et al. 2012). Combined with the fact that ∼30,000 21U-RNAs have been identified (Gu et al. 2012), 21U-RNAs can identify almost any foreign DNA sequence. Following target recognition by PRG-1, a second class of small RNAs (22G-RNAs) is produced, using the target transcript as a template (Das et al. 2008). In order to produce 22G-RNAs, presumably one of the RNA-dependent RNA polymerase (RdRP) enzymes of *C. elegans* is required as well as some genes known as mutator genes, like *mut-7* and *rde-3* (Bagijn et al. 2012). The 22G-RNAs are bound by a different set of Argonaute proteins, including the nuclear protein HRDE-1/WAGO-9 (Ashe et al. 2012; Buckley et al. 2012; Shirayama et al. 2012). The final outcome is reduced transcription and trimethylation of Lys9 of histone H3 (H3K9me3) of PRG-1 targeted genes (Ashe et al. 2012; Luteijn et al. 2012; Shirayama et al. 2012). Intriguingly, this transcriptional silencing response can acquire a PRG-1-independent, self-maintaining state. Under these conditions, HRDE-1, RDE-3, and MUT-7 are still required (Ashe et al. 2012; Luteijn et al. 2012; Shirayama et al. 2012).

The least-understood step in this PRG-1-mediated mechanism is how 21U-RNAs are made. In fact, we only know that 21U-RNA genes are marked by the presence of an upstream consensus motif (Ruby et al. 2006) of one transcription factor that plays a role in their transcription (Cecere et al. 2012) and that they are made as 5′-capped precursors that are at least 26 nucleotides (nt) in length (Gu et al. 2012). From these precursors, the two most 5′ bases are removed, likely followed by binding to PRG-1 and 3′ end trimming (Kawaoka et al. 2011). We do not know any of the factors acting during precursor processing. Here, we describe a forward mutagenesis screen aimed at the isolation of mutants that are piRNA-induced silencing-defective (Pid). This screen led to the identification of alleles of known PRG-1 pathway components but also revealed a novel protein, PID-1, that plays an important role in 21U-RNA biogenesis downstream from their transcription. We also describe genetic experiments revealing intriguing maternal and paternal effects of the PRG-1 pathway.

## Results and Discussion

### Isolation of mutants in the 21U pathway

In order to identify novel genes acting in the PRG-1-mediated silencing pathway, we made use of a strain expressing a GFP-Histone-2B fusion gene that carries a specific 21UR1-RNA recognition site in its 3′ untranslated region (UTR) (21U sensor) (Bagijn et al. 2012). We previously described how loss of HENN-1, the enzyme catalyzing the 3′ end methylation of 21U-RNAs, leads to partial desilencing of this transgene (Kamminga et al. 2012). We also demonstrated that this partially activated state is subject to spontaneous resilencing, after which this sensor construct is no longer responsive to removal of PRG-1 (Luteijn et al. 2012). In order to identify genes that act at any stage of the piwi pathway, we mutagenized a population of animals carrying a partially activated 21U sensor in a *henn-1* mutant background with EMS and scored for full activation of the 21U sensor (Supplemental Fig. S1A). Genome-wide resequencing of retrieved mutant strains identified premature stop mutations in *mut-7* and *rde-3* (Supplemental Fig. S1B). We also identified an interesting allele (*xf25*) of *hrde-1* carrying a nonsynonymous mutation affecting the last amino acid of HRDE-1 (A1032T). Given the important role for the most C-terminal residue of Argonaute proteins in making the binding pocket for the 5′ phosphate of the small RNA cofactor (Patel et al. 2006), we reasoned that this mutation might disrupt HRDE-1 activity. Indeed, a deletion allele of *hrde-1*, *tm1200*, was unable to complement *xf25* (data not shown), showing that HRDE-1(A1032T) is nonfunctional. In summary, our screen effectively identifies genuine components of the *C. elegans* piwi pathway.

### PID-1, a novel, general 21U-RNA biogenesis factor

Most of the alleles identified in our screen displayed normal levels of 21U-RNAs, as assessed by Northern blotting (Supplemental Fig. S1C), except for *xf14*. The locus affected by *xf14* was named *pid-1*. To probe the generality of the apparent 21U accumulation defect, we analyzed small RNAs from both wild-type and *pid-1(xf14)* young adult animals that also carry the 21U sensor through deep sequencing (Supplemental Table S1). Both libraries have a very similar size and display similar microRNA (miRNA) profiles (Supplemental Table S1; Supplemental Fig. S2A), indicating that the libraries can be well compared.

**Figure 1.**
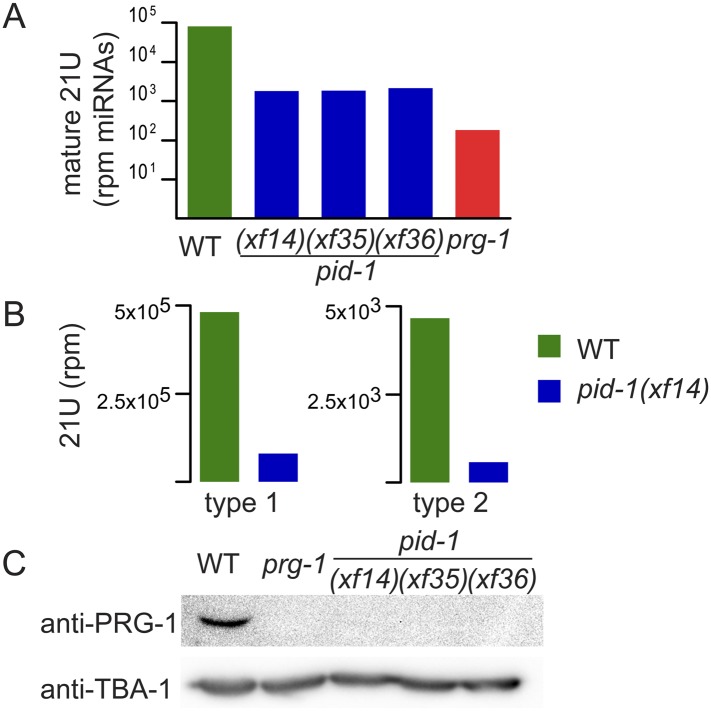
*pid-1* affects 21U-RNAs and PRG-1. (*A*) Bar diagram displaying normalized mature 21U read counts in strains with the indicated genotypes. Normalization was done to total miRNA read counts. Mature 21U reads were defined as those reads starting at the annotated mature 5′ end. (*B*) Bar diagram displaying type 1 and type 2 21U-RNA abundance in the indicated strains. Read counts reflect 21U counts obtained from NaIO_4_-oxidized small RNA populations normalized to total mapped non-rRNA reads. (*C*) Western blotting analysis of extracts from wild-type, *prg-1*, and *pid-1* mutant animals (young adults). Tubulin was used as a loading control.

As expected, in wild-type animals, we could detect small RNAs mapping to the 21U sensor (Supplemental Fig. S2B). These small RNAs display all characteristics of 22G-RNAs (Supplemental Fig. S3). While in *pid-1(xf14)* mutants these small RNAs still display all 22G characteristics, their levels are strongly reduced (Supplemental Fig. S2B).

We next checked mature 21U-RNA levels, normalizing to total miRNA counts. We found that, overall, 21U-RNA levels were 10-fold reduced in *pid-1(xf14)* mutants (Fig. 1A) and that most 21U-RNA loci responded similarly to *pid-1(xf14)* (Supplemental Fig. S2A). The loss of 21U-RNAs is slightly less than observed in *prg-1(pk2298)* mutants (Fig. 1A), suggesting that 21U-RNA biogenesis can proceed partially in the absence of PID-1. The 5′ nucleotide composition of the remaining 21U-RNAs in the *pid-1* mutants was still heavily biased toward uracil (Supplemental Fig. S2C).

Finally, we asked whether PID-1 equally affects both type 1 and type 2 21U-RNAs. Most 21U-encoding loci are characterized by a specific sequence motif upstream of the transcription start site (Ruby et al. 2006), and a later study showed that this motif may be a transcription factor-binding site (Cecere et al. 2012). However, it was shown recently that many loci exist that do not have this sequence motif and still produce 21U-RNAs. These two different classes have been referred to as type 1 and type 2 21U-RNAs, respectively (Gu et al. 2012). We found that *pid-1(xf14)* leads to a strong decrease in type 1 as well as type 2 21U-RNAs (Fig. 1B), indicating that PID-1 does not affect recognition of the upstream 21U sequence motif. We conclude that PID-1 is a factor involved in global 21U-RNA biogenesis.

### PID-1 affects PRG-1 levels and inhibits RNAi

The only gene thus far known to be required for 21U-RNA accumulation is *prg-1*. Hence, PID-1 could be required for transcription of *prg-1*. In order to test this, we performed RT-qPCR on wild-type and *pid-1* mutant animals (Supplemental Fig. S4A). This revealed that transcript levels of *prg-1* are unaffected by *pid-1*. In contrast, PRG-1 protein levels were strongly reduced upon mutation of *pid-1* (Fig. 1C), indicating a post-transcriptional effect of *pid-1* on PRG-1. Although we formally cannot exclude a role for PID-1 in PRG-1 translation, we interpret this result as instability of PRG-1 in the absence of 21U-RNAs, much like *Drosophila* Piwi is unstable without piRNAs, and Argonaute proteins are destabilized by loss of miRNAs (Olivieri et al. 2010; Smibert et al. 2013).

Since many PRG-1 pathway components affect RNAi triggered by exogenous dsRNA, we also probed the RNAi sensitivity of *pid-1* mutants. Using *pos-1* RNAi by feeding, we found that *pid-1* mutants, like *prg-1* mutants, are slightly hypersensitive (Supplemental Fig. S4B). Most likely, this reflects competition for components shared by the exo-RNAi machinery and the *prg-1* pathway, such as MUT-7 and RDE-3.

### Identification of pid-1 as F18A1.8

**Figure 2.**
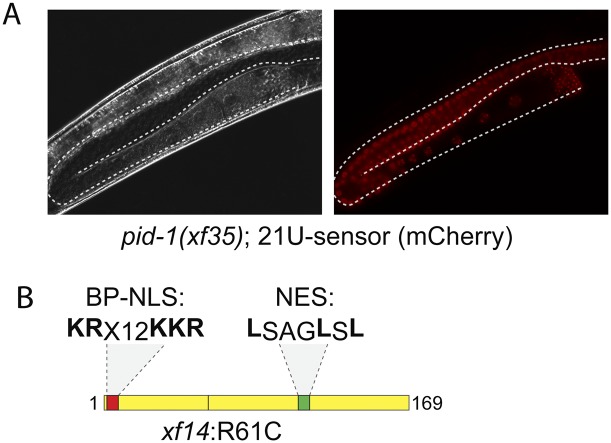
Identity of *pid-1*. (*A*) A typical *pid-1(xf35)* mutant animal carrying a mCherry-21U sensor transgene that has been crossed in from a *mut-7(pk204)* mutant background. The depicted animal is homozygous wild type for *mut-7*. (*B*) Schematic of the PID-1 protein.

To identify the *pid-1* gene, we first crossed the *pid-1(xf14)* allele into a *henn-1* wild-type background. This showed that the loss of 21U-RNAs in *pid-1(xf14)* animals is *henn-1*-independent (Supplemental Fig. S5A). Genetic mapping revealed that *pid-1* is genetically closely linked to the 21U sensor transgene on chromosome II (data not shown), and genome-wide resequencing revealed three nonsilent mutations in the area where *pid-1* was mapped; these three mutations affect F18A1.8, ZK1320.5, and F37H8.3. We then selected recombinants that separated the 21U sensor from these three mutations. Only recombinants that picked up a wild-type copy of F18A1.8 produced 21U-RNAs (Supplemental Fig. S5A), suggesting that F18A1.8 is *pid-1*. To test this, we generated two additional alleles (*xf35* and *xf36*) of F18A1.8 using CRISPR technology and probed the effect of these alleles on the activity of our 21U sensor and on 21U-RNA biogenesis. First, *xf35* and *xf36* do not complement *pid-1*(*xf14)* (Supplemental Fig. S5B; data not shown). Second, *pid-1(xf35)* was unable to silence a 21U cherry sensor transgene coming from a *mut-7(pk204)* mutant background (Fig. 2A). Third, both *xf35* and *xf36* have a similar effect on mature 21U-RNA levels as *pid-1(xf14)*, as revealed by deep sequencing (Fig. 1A). Finally, like in *pid-1(xf14)* mutants, PRG-1 levels are strongly reduced in *xf35* and *xf36* mutant extracts (Fig. 1C). Taken together, these data prove that *pid-1* corresponds to F18A1.8.

**Figure 3.**
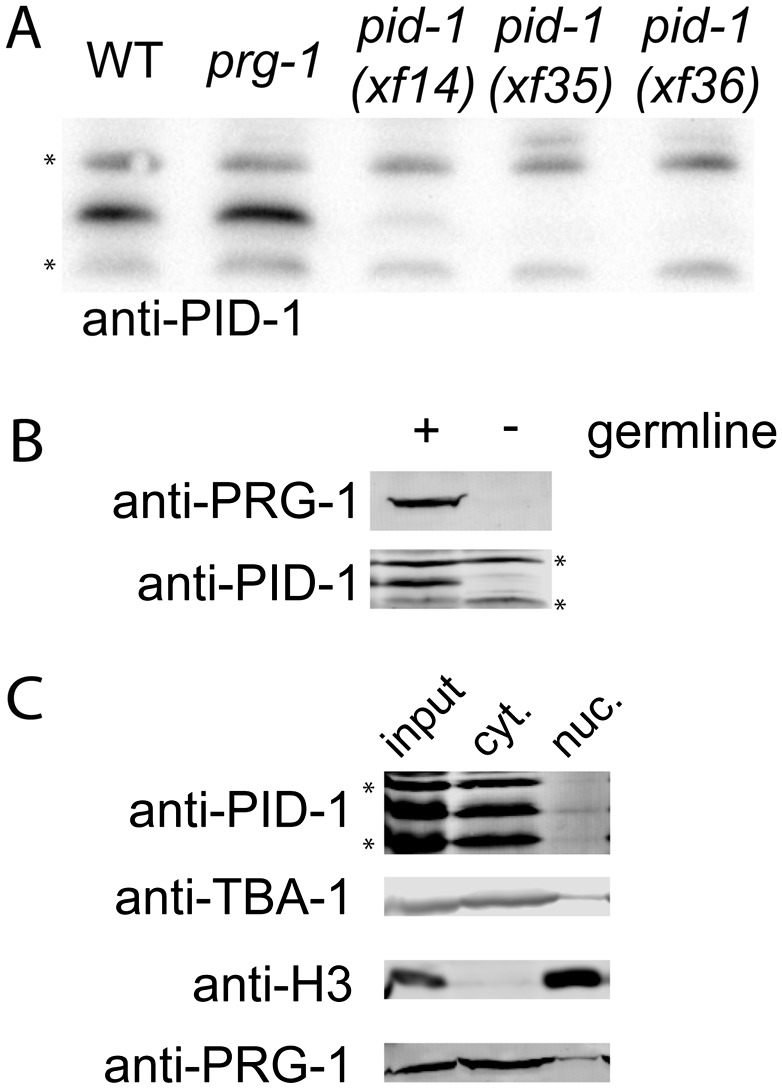
PID-1 is a mostly cytoplasmic, germline-expressed protein. (*A*) Western blot for PID-1 in the indicated backgrounds. The asterisks mark nonspecific signals from the custom-raised antiserum. The nonspecific signals were also used as a loading control. (*B*) Western blot detecting PID-1 and PRG-1 protein in *glp-4(bn2ts)* mutant animals grown at permissive (with germline) and restrictive (without germline) temperatures. The asterisks mark nonspecific signals from the custom-raised antiserum. (*C*) Western blot analysis of a subcellular fractionation experiment. TBA-1 was used to validate cytoplasmic fractions (cyt), and Histone H3 was used to validate nuclear fractions (nuc).

PID-1 is predicted to be a protein of 169 amino acids, and *pid-1(xf14)* results in an arginine-to-cysteine change at position 61 (R61C) (Fig. 2B). This mutation somehow affects PID-1 stability, since Western blotting reveals that PID-1 expression is strongly reduced in *pid-1(xf14)* mutant extracts, while the *xf35* and *xf36* alleles do not produce detectable PID-1 protein at all (Fig. 3A). Next, we tested whether PID-1, like PRG-1, is expressed specifically in the germline by performing Western blot analysis on mutants with a temperature-sensitive allele of *glp-4*, resulting in a lack of germline tissue in animals grown at 25°C. This revealed that PID-1 is expressed predominantly in the germline. This is consistent with published data showing that *pid-1* transcripts are selectively enriched in the primordial germ cells of *C. elegans* (Spencer et al. 2011).

No obvious domain structure is detected through regular BLAST analysis. However, at the N terminus, a bipartite nuclear localization signal may be present (**KR**EFSHITLASTPF**KKR**), and the protein may contain a nuclear export signal (LSAGLSL) as well (Fig. 2B), suggesting that PID-1 may be cycling between the nucleus and cytoplasm. Subcellular fractionation experiments suggest that the majority of PID-1 is cytoplasmic (Fig. 3C). However, given that the PID-1 signal is retained better in the nuclear fraction than the non-PID-1-specific signals, it is possible that a small part of the PID-1 pool may be associated with or be inside the nucleus (Fig. 3C).

### PID-1 does not affect 21U-RNA methylation

To further probe at which level PID-1 affects 21U-RNA biosynthesis, we NaIO_4_-oxidized small RNA. This procedure enriches for small RNA species that carry a 2′-O-methyl modification at their 3′ end, such as mature 21U-RNAs in *C. elegans* (Ruby et al. 2006), and it is believed that this methylation step finalizes 21U-RNA biogenesis. To assess the effect of the oxidation, we compared the ratio of 21U with miRNA reads in the tobacco alkaline phosphatase (TAP)-treated and the oxidized RNA-derived libraries. Using this analysis, we were unable to detect a significant difference between the wild type and *pid-1* mutants (Supplemental Table S1), indicating that the remaining 21U-RNAs in *pid-1(xf14)* mutants are still 2′-O-methylated. Furthermore, the global requirement of *pid-1(xf14)* for 21U-RNA accumulation is also observed in the libraries made from oxidized RNA (Supplemental Fig. S6A).

We also checked whether the step right before methylation is affected by PID-1. This step involves the trimming of the 21U 3′ end, presumably by an exonuclease. To assess this, we profiled the length distribution of those 21U reads from the TAP-treated libraries whose 5′ ends match precisely onto an annotated 5′ end of a mature 21U-RNA, indicating that the 5′ end has been processed (see below). This revealed no differences between the wild-type and mutant 21U-RNAs (Supplemental Fig. S6B). We conclude that PID-1 does not affect the 3′ end processing of 21U-RNAs.

### PID-1 acts during 21U precursor processing

**Figure 4.**
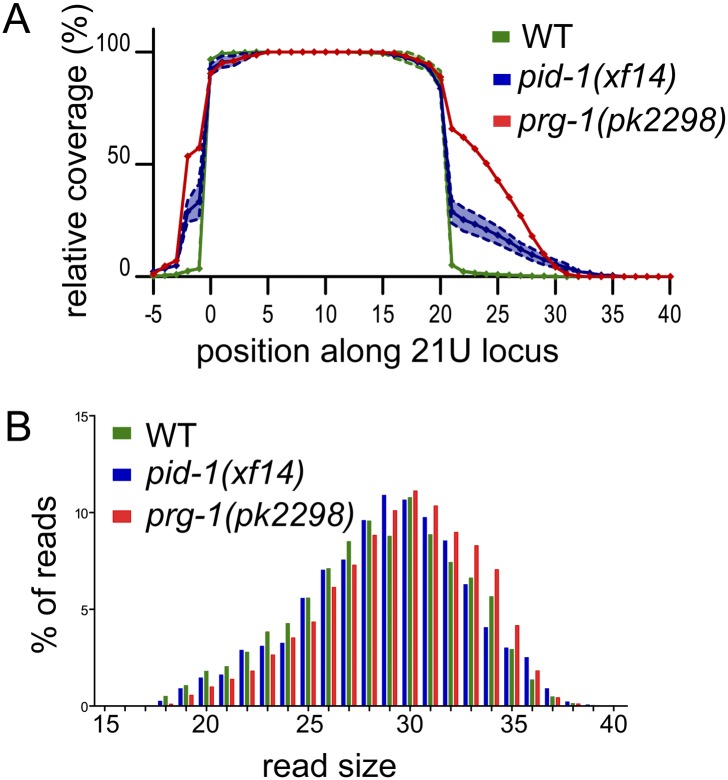
PID-1 acts during 21U precursor processing. (*A*) Relative coverage of individual bases of 21U loci by obtained cDNA reads from *prg-1, pid-1*, and wild-type libraries made from TAP-treated small RNA. The annotated 5′ ends of the analyzed loci are at position 0. The dotted lines reflect the standard deviation from the two wild-type samples and the three *pid-1* alleles. (*B*) A length distribution plot for 21U-RNA precursor reads obtained from the CIP-TAP-treated libraries.

We also probed our libraries for potential 21U-RNA precursor transcripts. It has been suggested before that these precursors mostly start 2 nt upstream of the 5′ end of the mature 21U-RNA, are 5′-capped, and are at least 26 nt in length (Gu et al. 2012). These species should be represented in our TAP-treated libraries, as TAP has decapping activity. Hence, we analyzed all reads that come from unique 21U loci (Supplemental Table S2) and start within 5 nt of the annotated mature 5′ end. In order to prevent strong biases by heavily expressed individual 21U loci, we restricted our analysis to those 21U loci that individually produce <1% of the 21U reads. We then plotted read coverage over these 21U loci, starting at position −5 and extending until position +40, with the start of the annotated mature 21U-RNA at position 0. First, such analysis in two replicate samples of wild-type animals reveals that this approach yields very reproducible patterns (Supplemental Fig. S6C). We then performed the same analysis on *prg-1(pk2298)* small RNA-derived cDNA libraries. This revealed that in the absence of PRG-1, ∼80% of the reads derived from 21U loci correspond to precursor transcripts, starting mostly at position −2 and extending to ∼35 nt in length (Fig. 4A). We then analyzed the effects of the three *pid-1* alleles (Fig. 4A; Supplemental Fig. S6C), revealing a clear accumulation of 21U-RNA precursors in animals lacking PID-1. Interestingly, the effect of *pid-1* is somewhat less than that of *prg-1*. Given the fact that *pid-1* mutants still produce low levels of 21U-RNAs while *prg-1* mutants almost lack them completely, the simplest interpretation of these data is that in *pid-1* mutants, 21U precursors can still be partially processed into mature 21U-RNAs. Finally, we profiled the length and locus coverage of 21U precursor transcripts in the diverse wild-type and mutant backgrounds using a protocol that selects for capped transcripts (Gu et al. 2012). This experiment did not reveal significant differences in the 21U precursor structure between any of the tested strains, suggesting that PID-1 does not affect the structure of 21U precursor transcripts (Fig. 4B).

### Genetic requirements for pid-1 and prg-1 during transgene (re)silencing

Finally, in order to better understand the global role of 21U-RNAs during the process of transgene silencing, we assessed how active transgenes from 21U-defective backgrounds react to the re-establishment of the 21U pathway. For that, we crossed *pid-1(xf14)*;21U sensor males into either wild-type or *pid-1* mutant hermaphrodites. As expected, offspring of these males mated with *pid-1*-defective hermaphrodites show strong transgene activity. Interestingly, however, when mated to wild-type hermaphrodites, these males sire offspring with active transgenes, albeit the fluorescence intensity is notably lower (Table 1). We observed similar transgene activity in offspring from *prg-1(pk2298)*;21U sensor males mated with wild-type hermaphrodites. Full silencing of the transgene is only observed in the next generation (Table 1). This shows that while 21U-RNAs start to silence a novel target within one generation, full silencing can be achieved only upon joint passage through the germline. Possibly, 22G-RNA levels need to accumulate in order to establish full silencing.

**Table 1.**
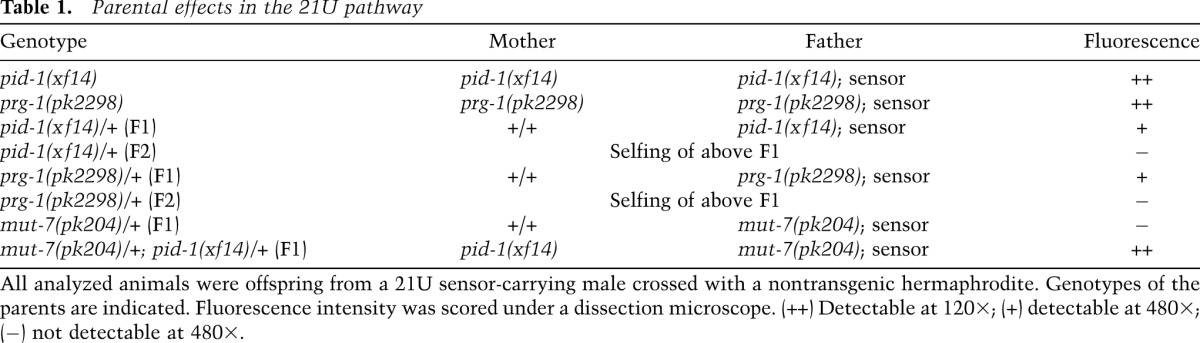
Parental effects in the 21U pathway

Next, we crossed an active 21U sensor from *mut-7(pk204)* males into both wild-type and *pid-1* mutant hermaphrodites and analyzed transgene expression in the F1. This revealed a very strong maternal effect of 21U-RNAs: The wild-type hermaphrodites produced fully silenced offspring, while the progeny of the *pid-1* mutant mothers could not initiate detectable silencing even though they were heterozygous wild type for *pid-1*. This result highlights at least two properties of the 21U pathway. First, the maternally provided 21U-RNA pool is essential to establish resilencing of a *mut-7(pk204)*-activated transgene, suggesting that the initiation of silencing by 21U-RNAs happens during embryonic development using maternally provided 21U-RNAs. Second, a *mut-7*-activated transgene coming in via the sperm is easier to resilence by 21U-RNAs than a *pid-1* or a *prg-1*-activated transgene. We conclude that some silencing information must be present in *mut-7*-derived sperm but is lacking in *prg-1*-defective sperm and that this information can be efficiently used by the maternal 21U-RNA pool.

### Conclusions

We identified a novel gene, *pid-1*, that plays an important role during 21U-RNA biogenesis in *C. elegans*. PID-1 is a mostly cytoplasmic protein that likely acts during the processing of 21U-RNAs downstream from their transcription. The mature 21U-RNAs that are still made in *pid-1* mutants are indistinguishable from wild-type 21U-RNAs, and, relative to these mature 21U-RNAs, 21U-RNA precursors accumulate upon loss of PID-1. Furthermore, the structure of 21U precursor transcripts is not affected by PID-1. Given these results as well as the potential nuclear import and export signals on PID-1, we suggest that PID-1 may somehow guide 21U precursors to a site where they are further processed into mature 21U-RNAs. We note that in other species, like mice or *Drosophila*, PID-1 orthologs cannot be identified through BLAST analysis. This may be related to the fact that, overall, the 21U-RNA pathway in *C. elegans* deviates significantly from piRNA pathways in other animals. However, given the still poor characterization of piRNA biogenesis in general, proteins with similar functions but with diverged sequences may operate in mammalian or fly piRNA biogenesis.

We found that *pid-1* as well as *prg-1* partially inhibit RNAi triggered by exogenous dsRNA. Much like what is described for other RNAi-hypersensitive mutants, we attribute this to the fact that the *prg-1* pathway included proteins shared with the exo-RNAi pathway (Duchaine et al. 2006; Pavelec et al. 2009), and thus ongoing *prg-1* activity in *C. elegans* puts a significant claim on those shared resources.

On the genetic level, we showed that the initiation of transgene silencing by 21U-RNAs depends on maternally provided 21U-RNAs. These findings are consistent with the maternal contributions of piRNA populations observed in other species, like *Drosophila* (Brennecke et al. 2008) and zebrafish (Houwing et al. 2007), and highlight the important role for maternally provided small RNA populations in order to establish silencing in offspring. In addition, we found that the genetic background of the sperm can have an effect on the silencing of a locus. Considered together, parental influences play important roles during the establishment of the epigenetic state of an allele through the 21U pathway in *C. elegans*.

## Materials and methods

### *C. elegans* strains

*C. elegans* was cultured on NGM plates, with *Escherichia coli* OP50 as a food source. Alleles that were used in this study are listed in the Supplemental Material.

### CAS9-mediated disruption of F18A1.8

To generate additional mutations in F18A1.8, we targeted a site in the first exon (GGAGTTTTCGCATATTACTT) with CRISPR/Cas9 as previously described (Waaijers et al. 2013). The sequences of *pid-1* at the targeted site and of the two derived alleles are listed in the Supplemental Material.

### Microscopy

The screen for Pid mutants was performed using a Zeiss M2Bio dissecting microscope. Fluorescence images were taken on a Leica DM6000 B upright microscope with 400× amplification. Silencing onset of active transgenes was analyzed using a Leica M165 FC stereo microscope with a 1× objective (120× amplification) or a 4× objective (480× amplification).

### RNAi

Sensitivity to RNAi was tested by feeding *E. coli* expressing dsRNA against *pos-1* (Ketting et al. 1999). Animals were placed on RNAi food as L4 larvae and allowed to lay eggs for 2 d, after which survival was scored. Each strain was tested between four and six times.

### Data analysis

Detailed information on data analysis is provided in the Supplemental Material.

### PID-1 antibody

Custom, affinity-purified antibodies against PID-1 were ordered from SDIX, using the whole protein sequence of PID-1 as an antigen. The antibody (animal no. Q5941) was used in a 1:200 dilution on Western blots.

### Nucleo–cytoplasmic fractionation

Synchronized adult worms were washed with extract buffer (20 mM MOPS at pH 7.5, 40 mM NaCl, 90 mM KCl, 2 mM EDTA, 0.5 mM EGTA, 10% glycerol, 2 mM DTT, proteinase inhibitors [Roche Complete ULTRA]) and resuspended to a final volume of 4 mL. The suspension was then dripped into liquid nitrogen, and the frozen pellets were ground to a fine powder. The powder was transferred to a glass douncer, thawed on ice, and sheared (30 strokes, piston B). The extracts were then centrifuged twice at 200*g* to remove debris. The supernatant (crude extract) was centrifuged at 2000*g* to separate the nuclear fraction from the cytoplasmic fraction. Nuclear fractions were washed twice with extract buffer, whereas the cytoplasmic fraction was centrifuged at 21,000*g* to remove any remaining debris.

### Small RNA library preparation

Small RNA was isolated with a Mirvana kit from Life Technologies. Following isolation, this RNA was treated with TAP and calf intestinal phosphatase followed by TAP (CIP-TAP) or NaIO_4_. The wild-type;21U sensor and *pid-1(xf14)*;21U sensor libraries (oxidized and nonoxidized) were prepared as described before (Kamminga et al. 2012). Wild-type, *prg-1*, *pid-1(xf14)*, *pid-1(xf35)*, and *pid-1(xf36)* libraries (TAP and/or CIP-TAP) were prepared as described in the Supplemental Material. Sequencing data are available at Gene Expression Omnibus, entry GSE55309.
